# Recurrent Graves' Disease Following Near-Total Thyroidectomy: A Case Report and Literature Review

**DOI:** 10.7759/cureus.52260

**Published:** 2024-01-14

**Authors:** Lakshmi P Menon, Syeda Naqvi, Jhansi Maradana, Dinesh Edem

**Affiliations:** 1 Endocrinology, Diabetes and Metabolism, University of Arkansas for Medical Sciences, Little Rock, USA; 2 Endocrinology, Diabetes and Metabolism, Mass General Brigham Wentworth-Douglass Hospital, Dover, USA

**Keywords:** graves’ disease, thyroglobulin, tsh receptor antibody, thyroid eye disease, thyroid surgery

## Abstract

Recurrent Graves' disease due to regrowth of thyroid tissue is a rare complication of near-total thyroidectomy, which can be challenging to recognize and manage. Here, we present the case of a 30-year-old woman with Graves' disease and thyroid eye disease who underwent near-total thyroidectomy with resultant hypothyroidism. Her levothyroxine dose requirement gradually decreased and thyroglobulin level increased, which led to the diagnosis of recurrent Graves' disease. A neck ultrasound showed regrowth of thyroid tissue. The treatment options in such cases are repeat thyroid surgery and radioactive iodine ablation. The patient had moderate-severe active thyroid eye disease, so radioactive iodine ablation was contraindicated. Repeat surgery was deemed high risk due to the location of the residual thyroid tissue near the recurrent laryngeal nerve. Watchful waiting with serial thyrotropin (TSH) receptor antibody monitoring was chosen, and her levothyroxine dose was adjusted based on her thyroid function tests. There was a normalization of her TSH receptor antibody level over the next two and half years and stabilization of levothyroxine dose requirement. Recurrent Graves' disease must be considered when there is an ongoing decrease in the levothyroxine dose requirement associated with a rise in the serum thyroglobulin level following near-total thyroidectomy. Conservative management with medical therapy can induce remission in the case of recurrent Graves' disease following near-total thyroidectomy, without the need for radioactive iodine ablation or repeat thyroid surgery.

## Introduction

Graves’ disease is an autoimmune disorder in which there are antibodies directly against the thyroid follicular cells. The main autoantigen in Graves’ disease is the thyroid-stimulating hormone receptor antibody (TRAb), which binds to the thyrotropin receptor. TRAb generally has a stimulatory effect and leads to the oversecretion of thyroid hormones and proliferation of thyrocytes, resulting in hyperthyroidism [1]. Graves’ disease can be diagnosed by the finding of elevated TRAb in a patient with hyperthyroidism or by the presence of a diffusely increased uptake on a thyroid uptake and scan [2]. The treatment options for Graves’ hyperthyroidism include antithyroid medications (ATDs), radioactive iodine ablation (RAI), and total or near-total thyroidectomy [2]. Treatment with antithyroid medications can induce remission in around 50% of cases [3]. Both RAI and total thyroidectomy usually lead to the development of hypothyroidism requiring lifelong levothyroxine replacement to maintain euthyroidism.

In this context, we present a case of a 30-year-old woman with Graves’ disease who underwent near-total thyroidectomy that was complicated by the regrowth of thyroid tissue due to a TRAb stimulation. She had progressive reduction in the levothyroxine dose requirement leading to the diagnosis of recurrent Graves’ disease.

## Case presentation

A 30-year-old woman with a past medical history of depression, anxiety, and Crohn’s disease presented to the ER in October 2019 with a four-month history of palpitations, weight loss despite increased appetite, shortness of breath, worsening anxiety, diplopia, and prominence of eyes. She was seven months postpartum at that time and had initially attributed her symptoms to being postpartum. On physical examination, her heart rate was 123, respiratory rate was 26, and her BMI was 38.70 kg/m^2^. She had a diffusely enlarged tender goiter, tachycardia, and fine tremors. The estimated thyroid size was 60 grams. Eye exam revealed lid lag, periorbital edema, proptosis, and chemosis, consistent with thyroid eye disease (TED). Labs revealed that the TSH was suppressed at <0.02 ng/dL (0.58-1.64 ng/dL), free T4 was elevated to 2.76 mg/dL (0.58-1.64 ng/dL), and total T3 elevated to 4.0ng/mL (0.7-2.0 ng/mL). The TRAb was elevated at 2.76 ng/dL (<1.75 IU/L), and thyroid-stimulating immunoglobulin (TSI) was elevated at >500% (<=122%), diagnostic of Graves’ disease. Thyroid ultrasound showed thyromegaly with the right lobe measuring 7.1 x 3.2 x 3.5 cm and the left lobe measuring 6.9 x 3.2 x 2.8 cm. No nodules were noted. Methimazole 20 mg twice daily, propranolol 40 mg four times a day, and prednisone 60 mg daily were started. She reported chronic tobacco use and was counselled about smoking cessation.

**Figure 1:**
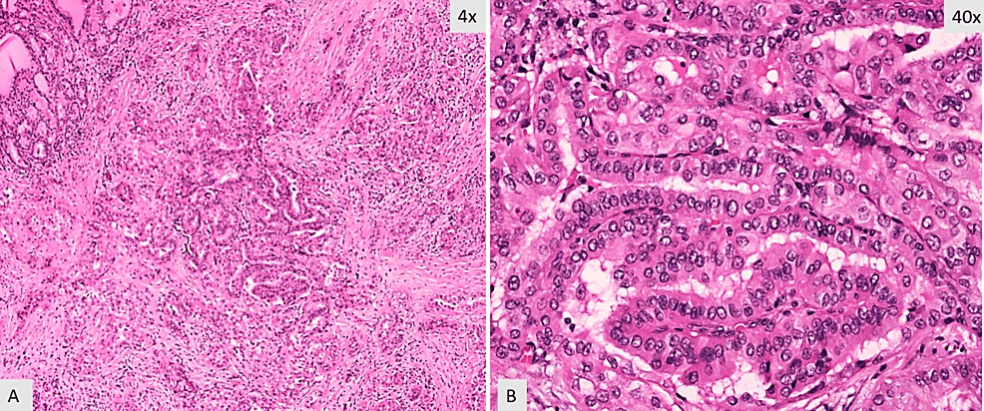
Histology from the total thyroidectomy showing the focus of the papillary thyroid carcinoma A. Papillary thyroid thyroid carcinoma, classic variant in the background of papillary hyperplasia (hematoxylin & eosin stain, 4X magnification). B. Papillary thyroid carcinoma with nuclear enlargement and overlapping. Optically clear nuclei with thick nuclear membrane (hematoxylin & eosin stain, 40X magnification).

Over the next two months, she developed worsening neck swelling and new onset dysphagia. Thyroid examination revealed an increase in the size of the goiter with an estimated thyroid size of 90 grams. She was referred to ENT who performed a total thyroidectomy in December 2019. The surgery was complicated by the high vascularity of the gland, due to which residual tissue was left behind near the recurrent laryngeal nerve. Pathology showed a 7.8 x 4.5 x 4.2 cm right lobe, 2.0 x 1.4 cm isthmus, and 6.2 x 3.6 x 3.2 cm left lobe, and the capsule was predominantly intact. Diffuse papillary hyperplasia was noted, consistent with the diagnosis of Graves’ disease. A 0.4 cm focus of papillary thyroid cancer was noted on the left lobe (Figure 1A, 1B).

Given that the micro-papillary thyroid carcinoma was staged at pT1aNxMx, no further surveillance or RAI ablation was performed. The staging of pT1aNxMx denotes that the primary tumor was less than 1 cm in size and limited to the thyroid and regional lymph nodes or the presence of distant metastasis was not assessed on this pathology specimen. 

**Figure 2:**
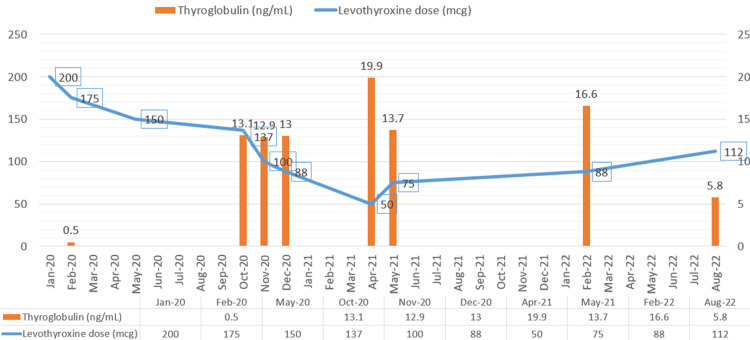
Relationship between the thyroglobulin level, TRAb, and levothyroxine dosage

She was started on levothyroxine 200 mcg daily following near-total thyroidectomy. TSI (>500%, normal value <122%) and TRAb (>18.44IU/L, normal value <=1.75IU/L) remained high one month after the surgery. The post-operative thyroglobulin was 0.5 ng/mL, which indicated that minimal residual thyroid tissue was left behind during the surgery. Serial labs showed persistent hyperthyroidism due to which her levothyroxine dose was gradually lowered until she reached a nadir dose of levothyroxine 50 mcg daily at one year after her near-total thyroidectomy (Figure 2). Of note, her weight based levothyroxine requirement was 212 mcg daily at that time. Her thyroglobulin level increased from 0.5 ng/mL two months after the surgery to 19.9 ng/mL one year later.

**Figure 3:**
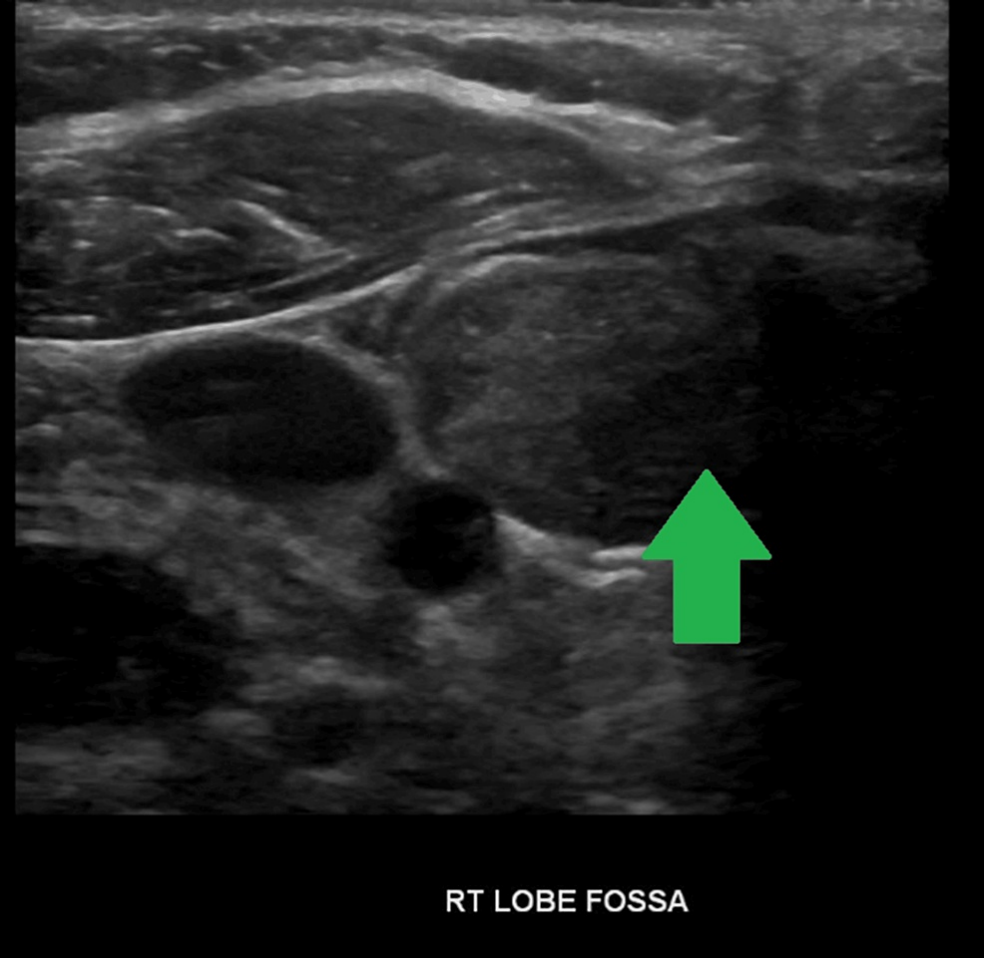
Neck ultrasound showing regrowth of thyroid tissue

In September 2020, she went to the ER for throat pain following ingestion of a pizza roll and concern for food getting stuck in her throat. CT neck soft tissue with contrast was obtained, which showed a hyperdense nodule in the region of the right thyroid lobe measuring 2.5 x 1.9 x 1.9 cm, concerning for the regrowth of thyroid issue. A neck ultrasound was obtained, which confirmed this finding (Figure 3).

The recurrence of micro-papillary thyroid cancer was unlikely because the tumor was present on the left lobe at the initial surgery. Thyroid uptake and scan was not obtained since the ultrasound finding was consistent with the regrowth of thyroid tissue. She was seen by surgery who deemed her a poor candidate for repeat surgery due to the location of the thyroid tissue in close proximity to the recurrent laryngeal nerve placing her at high risk for recurrent laryngeal nerve injury. RAI was contraindicated due to the presence of moderate-severe active thyroid eye disease. Following a multidisciplinary discussion with endocrinology, surgery, and ophthalmology, the decision was made to continue conservative management.

On follow-up visit in May 2021, her TSH was elevated to 4.71, due to which her levothyroxine dose was increased to 75 mcg. She displayed a gradual increase in her levothyroxine dosage requirement over the next year along with a slow decline in her thyroglobulin level (Figure 3).

At her most recent clinic visit in August 2022, she was maintained on levothyroxine 112 mcg daily. Her TRAb has declined into the normal range at 1.65 IU/L, and her thyroglobulin has decreased to 5.8 ng/mL, consistent with reduced endogenous thyroid hormone production. Neck ultrasound in July 2022 showed a decrease in the size of the residual thyroid tissue, which now measures 2.5 x 1.2 x 1.6 cm. This decrease in the size of the thyroid tissue is secondary to the decreased stimulation of the thyrocytes due to the normalization of TRAb and implied that there would be no further increase in thyroid hormone production from the residual thyroid tissue.

She was found to have active TED with a clinical activity score (CAS) of 3 immediately prior to her near-total thyroidectomy. CT of the orbits showed bilateral proptosis with enlarged extraocular muscles with mild to moderate crowding at the bilateral orbital apex. She displayed a worsening of her TED after total thyroidectomy with an increase in CAS to 7 in May 2020 despite a course of oral prednisone 40 mg daily. In addition, she displayed a worsening of her mood disorder due to the use of high-dose glucocorticoids due to which IV pulse methylprednisolone was not administered. She was subsequently started on teprotumumab every three weeks and completed a total of seven doses. Teprotumumab is a monoclonal antibody that acts as an antagonist at the insulin-like growth factor-1 receptor and attenuates the inflammatory response in TED. Her CAS improved to 2. At her most recent visit, her TED was inactive, but she had residual proptosis. Ophthalmology plans to see her in clinic every six months to monitor for signs of recurrence of active TED. They plan to perform orbital decompression to treat the proptosis if there is no recurrence of active TED.

## Discussion

This case highlights a rare complication of near-total thyroidectomy for the treatment of Graves’ disease and provides an overview of its diagnosis and treatment. The patient had a progressive decrease in her levothyroxine dose post near-total thyroidectomy, which raised the concern for regrowth of residual functional thyroid tissue. Imaging studies were consistent with regrowth of thyroid tissue. This case also highlights the utility of serum thyroglobulin for serving as a marker for thyroid hormone synthesis in the case of a benign thyroid disorder.

Thyroidectomy is the preferred treatment for Graves’ disease under certain select situations, such as the presence of large thyroid nodules, thyromegaly with compressive symptoms, suspected or confirmed malignancy, or coexisting primary hyperparathyroidism [2]. Thyroidectomy can also be performed for treating Graves’ disease in the presence of moderate-severe active TED. A prospective study showed that both subtotal and total thyroidectomy lead to an improvement in CAS in patients with moderate-severe active TED [4].

The optimum extend of thyroid surgery in Graves’ disease has been a matter of debate historically. Bilateral subtotal thyroidectomy, which involves leaving a 2-4 gram remnant of thyroid tissue bilaterally was initially favored because of the aim of avoiding surgical hypoparathyroidism and recurrent laryngeal nerve injury [5]. This approach carries the risk of causing recurrent hyperthyroidism, especially if a large thyroid remnant was left behind or if the TSH receptor antibody titer is high. The risk of persistent hyperthyroidism is around 30%, with permanent hypothyroidism in 60% and long-term euthyroidism in only 10% of the patients [6]. Unilateral subtotal thyroidectomy with contralateral hemithyroidectomy (Dunhill procedure) involves leaving a 4-7 gram remnant on the side of the recurrent laryngeal nerve and macroscopic resection of the entire thyroid tissue on the contralateral side. Total thyroidectomy involves resection of all visible thyroid tissue bilaterally. It reliably induces hypothyroidism and carries a lower risk of recurrent hyperthyroidism than subtotal thyroidectomy. The risks of surgical complications, including permanent hypoparathyroidism and recurrent laryngeal nerve injury, are similar between subtotal thyroidectomy and total thyroidectomy when performed by a high-volume thyroid surgeon.

A prospective, single-center study of patients with Graves’ disease who were randomized to bilateral subtotal thyroidectomy, Dunhill procedure, and total thyroidectomy showed that the TRAb titer decreased in all three groups equally and reverted into the normal range by the end of the study period in 76% of the study population [7]. There was an improvement in eye symptoms following surgery in 74% of the patients, no change in 21% of the patients, and worsening in 5% of the patients with no between-group differences. The post-operative complication rates of laryngeal nerve paralysis, bleeding, and permanent hypoparathyroidism did not show statistical significant differences between the three groups. The American Thyroid Association guidelines recommend total or near-total thyroidectomy as the procedure of choice for the surgical management of Graves’ disease [2].

Near-total thyroidectomy involves the removal of the entire thyroid gland except for a unilateral or bilateral remnant of less than 1 gram. A systematic review of total versus near-total thyroidectomy in Graves’ disease showed that near-total thyroidectomy was associated with a lower risk of hypoparathyroidism [8]. There was no increased risk of recurrent hyperthyroidism with near-total thyroidectomy as compared to total thyroidectomy.

The initial post-operative levothyroxine dose following total thyroidectomy is determined by the formula 1.6 mcg/kg body weight per day, which provides the full replacement dose [2]. This formula has been reported to overestimate the levothyroxine dose requirement in obese individuals because the levothyroxine dose requirement is mainly a function of the lean body mass. In one retrospective cohort study that looked into weight-based levothyroxine dose requirement in patients who underwent total thyroidectomy, the levothyroxine dose required to achieve euthyroidism was 1.28 mcg/Kg for BMI > 40 kg/m^2^ [9]. Our patient weighed 133.7 kg at the time of surgery with a BMI of 46.17 kg/m^2^ due to which her levothyroxine dose was estimated at 212 mcg daily (based on the formulate 1.6 mcg/Kg), and she was started on levothyroxine 200 mcg daily. The levothyroxine dose was gradually lowered based on persistent hyperthyroidism until she reached a nadir dose of 50 mcg daily (corresponding to 0.32 mcg/kg). The dose requirement is lower in individuals with hypothyroidism who have a partially preserved thyroid function, such as some individuals with Hashimoto’s thyroiditis [10]. The patient’s decreased need for levothyroxine relative to her body weight was indicative of persistent endogenous thyroid hormone production, and this was confirmed by the finding of the elevated thyroglobulin level.

The role of serum thyroglobulin to serve as a marker for biochemically recurrence of papillary thyroid cancer following total thyroidectomy is well established [11]. Our case demonstrates that the serum thyroglobulin is helpful in tracking the endogenous thyroid hormone production when there is evidence of recurrent Graves’ disease following total thyroidectomy. The level of thyroglobulin rose with the decrease in the levothyroxine dose requirement and subsequently declined when there was a normalization of the TSH receptor antibody level and increase in the levothyroxine dose requirement.

A neck ultrasound helps locate the site of the regrowth of thyroid tissue in the case of suspected recurrent or residual Graves’ disease. If the neck ultrasound does not show evidence of thyroid tissue, a thyroid uptake and scan should be obtained to evaluate for the presence of ectopic thyroid tissue. Recurrent or residual Graves’ disease and hyperthyroidism following thyroidectomy has been reported in ectopic thyroid tissue sites, namely, mediastinum [12,13], infrahyoid [14], thyroglossal duct remnants [15], lateral neck [16,17], or regrowth of thyroid tissue in the thyroid bed [18]. Therapeutic options include lowering the levothyroxine dosage, initiating ATDs [12,14], RAI ablation [12,14], or repeat surgery [14,15,16,17].

In our case, RAI ablation was contraindicated given the moderate-severe active TED. Repeat surgery for the resection of the residual thyroid tissue is associated with the risk of wound infection, laryngeal nerve paralysis, and hypocalcemia. We chose a conservative approach of a watchful observation with the lowering of the levothyroxine dose, monitoring TRAb and thyroglobulin levels. As the levels of TRAb improved to the normal range, the thyroglobulin level also reduced and levothyroxine requirements went up, without any additional therapy like ATDs, RAI, or repeat surgery. There was a resolution of the active TED along this time frame, which was attributed to the decrease in the TRAb level. TRAb binds to the TSH receptors on the orbital soft tissue and stimulates the release of pro-inflammatory cytokines. TRAb titers correlate with the disease severity in TED [19]. Hence, a decrease in the TRAb titer can lead to an improvement in TED.

## Conclusions

Recurrent Graves’ disease is a rare complication of near-total thyroidectomy for Graves’ disease, which should be suspected when there is a progressive decrease in the levothyroxine dose requirement along with a rise in the thyroglobulin level. A high preoperative TRAb titer is a risk factor for recurrent Graves’ disease by stimulating the proliferation of thyrocytes after near-total thyroidectomy. Conservative management with medical therapy can induce remission in the case of recurrent Graves’ disease following near-total thyroidectomy. Our case illustrates the importance of a watchful observation along with surveillance of TRAb and thyroglobulin levels in diagnosing and treating this condition. Total thyroidectomy should be preferred over near-total thyroidectomy to reduce the risk of developing recurrent Graves' disease due to regrowth of thyroid tissue.
